# Biomaterials and Therapeutic Agents in Dental Research: From Bioactive Materials to Locally Controlled and Precision Therapies

**DOI:** 10.3390/pharmaceutics18101203

**Published:** 2026-09-22

**Authors:** Pavel Seredin, Dmitry Goloshchapov, Tatiana Litvinova

**Affiliations:** 1 Department of Solid-State Physics and Nanostructures, Voronezh State University, 394018 Voronezh, Russia; 2 Department of Higher Mathematics, Voronezh State Pedagogical University, 394043 Voronezh, Russia

The field of dental biomaterials is growing far beyond simply replacing damaged tissues or providing the mechanical support needed for restorations. Just as important now are materials and drug-delivery platforms that can adapt to the constantly changing microenvironment of the oral cavity—suppressing harmful biofilms, supporting regeneration, enabling local delivery of therapeutic molecules, and, crucially, controlling exactly when and where these molecules act. This evolution is bringing dental materials science much closer to pharmaceutical technologies, microbiology, regenerative medicine, and quantitative modeling.

## 1. From the First to the Second Issue: From Functional Ideas to Integrated Therapeutic Systems

The first issue of the special edition *Biomaterials and Agents: Pharmaceutical and Biomedical Applications in Dental Research* made it clear how fast the field of dental biomaterials and therapeutic agents is expanding. In the 2024 concluding editorial, a thematic analysis pointed to several closely connected directions: nanomaterials and therapeutic agents, regeneration of bone and dental tissues, mineralization of hard dental tissues, anti-biofilm strategies, and the prevention and treatment of oral diseases [1]. Together, these areas formed a “map” of the key functional components of modern dental biomaterials science.

The second edition takes the next conceptual step. Rather than grouping these works mainly by the type of material or the specific agent used, it brings them together around a shared objective: controlling local effects in both time and space. Silver nanoparticles and photodynamic therapy are designed to work directly on the “site” of pathological biofilms; hydrogels can serve as both structural biomaterials and potential carriers for therapeutic substances; modeling local pharmacokinetics connects drug release to transport, local exposure, and biological outcome; functionalized implant coatings turn the material surface into an interface for delivering regulatory molecules; and targeted lipid nanoparticles extend this idea further by enabling tissue-specific delivery of nucleic acids [2,3,4,5,6,7].

These publications may not aim to cover the entire field exhaustively, but they do reflect a broader trend: the convergence of materials science, local drug delivery, targeting, and quantitative modeling. So, while the first edition primarily highlighted the variety of materials, agents, and biological functions, the second emphasizes the need to integrate them.

We see this shift as moving from a functional biomaterial toward a local therapeutic system—where the material (or carrier), active agent, microenvironmental state, spatiotemporal exposure, and biological response are treated as interdependent design parameters (Figure 1). The pharmaceutical perspective is especially important here. For local dental therapy, simply having the right active ingredient in the material—and even ensuring a particular release profile—is not enough to guarantee the desired therapeutic outcome. What really matters is the actual exposure, which depends on how well the system stays in the target area, how the drug travels through saliva, pellicle, and biofilm, how it interacts with mucosal and mineralized tissues, how quickly it is cleared locally, and how strongly it binds to the intended biological target.

## 2. Control of Dysbiotic Biofilms and Maintenance of Local Homeostasis

One of the biggest challenges in dentistry is dealing with microbial biofilms. Their spatial organization, interspecies interactions, features of the extracellular matrix, and the ability of microorganisms to adapt to harsh conditions all substantially weaken the impact of conventional antimicrobial strategies. Because of this, modern approaches are increasingly shifting away from the simple goal of “reducing microbial load” toward managing dysbiotic biofilms in a way that preserves or restores a stable relationship between the microbiota and host tissues.

The study by Pérez-Sáenz et al. [2] illustrates the potential of silver nanoparticles as a local antimicrobial option for endodontics. The authors tested particles of different sizes in in vitro and ex vivo models, using microbiological samples taken from patients with pulpal and periapical pathology. Their results showed antibacterial and anti-adhesive effects, with performance depending on both the properties of the sample and the exposure time. These findings support the promise of nanomaterials for influencing complex endodontic biofilms, while also underscoring the need to carefully optimize concentration, exposure duration, effect stability, and biocompatibility.

Another strategy is discussed in the review by Sousa et al. [3], which examines antimicrobial photodynamic therapy as an add-on to nonsurgical treatment of periodontitis. The idea of local photodynamic action is appealing because it could reduce reliance on systemic antibiotics. Still, the evidence from randomized studies is inconsistent: photosensitizers, light sources and doses, number of sessions, patient characteristics, and microbiological assessment methods differ widely. As a result, the technical promise of this approach has not yet eliminated the need for clinically meaningful standardized protocols.

Together, these studies suggest that evaluating new antimicrobial platforms should not be limited to how much individual microorganisms are suppressed. Greater priority should be given to how organized multispecies biofilms are affected, how long the effect lasts, how likely biofilm recolonization is, and how commensal microbiota and host tissues are impacted. Achieving this requires broader use of standardized multispecies models, ex vivo systems, and endpoints that reflect not only microbicidal activity but also restoration of local homeostasis.

## 3. Biomaterial as a Regenerative Matrix and Carrier

A second key direction is developing biomimetic and bioactive matrices that do two things at once: provide a supportive microenvironment for tissue regeneration and act as carriers for therapeutic components. This is especially important in the oral cavity, where materials must function reliably at the interface between hard and soft tissues while facing constant mechanical stress, as well as saliva, microbiota, and inflammatory factors [8,9,10].

The review by Aryal A. C. et al. [4] organizes research on dentistry-focused hydrogels based on cellulose derivatives. These systems offer adjustable mechanical properties, high water content, biodegradability, and the ability to load drugs or bioactive components. Potential applications include regeneration of dentin and pulp, periodontal tissues, alveolar bone, and local drug delivery. The review also highlights a major translational gap: much of the work remains at the in vitro stage, while comprehensive in vivo studies and clinical evidence are far less common. This pattern is typical for regenerative biomaterials—showing attractive function in the lab does not guarantee consistent performance in the real, dynamic, and individually variable oral microenvironment.

Therefore, for the next generation of materials, it is essential to evaluate not just one functional feature, but the full context of “material—interface—tissue—microbiota—mechanical and chemical load” [10].

## 4. From Release Profile to Control of Local Exposure

Simply having a drug present in a biomaterial does not guarantee effective local therapy. To achieve a clinical benefit, it is fundamentally important what concentration of the active substance actually reaches the target area, how long it remains there, how deeply the drug penetrates the biofilm or tissue, and how local physiology influences its transport and elimination.

This issue is directly addressed by Rakoczi et al. [5], who propose a quantitative framework for modeling local drug delivery in the oral cavity. Their approach links traditional drug-release kinetics models with a physiologically oriented mini-PBPK-type model, along with local pharmacokinetic–pharmacodynamic relationships. Special attention is given to processes that are often underrepresented in standard systemic pharmacokinetics: adsorption onto the acquired pellicle, diffusion through biofilm, exchange between saliva, mucosa, and the periodontal pocket, and drug removal by gingival fluid.

The value of this approach extends beyond modeling specific existing periodontal drug formulations. Mechanistic models could become tools for designing the biomaterials and carriers themselves. Instead of empirically selecting the highest loading or the longest release, it becomes possible first to define the therapeutic window and local exposure profile that are needed, and then optimize material properties, geometry, degradation rate, and delivery kinetics to match that goal. In other words, the optimization focus shifts away from the release profile itself and toward the local exposure profile.

A particularly important next step is adding patient-specific parameters such as salivary flow rates, gingival fluid secretion intensity, periodontal pocket depth, biofilm structure, inflammatory status, and local anatomical features. At that point, model-informed drug-platform design naturally aligns with personalized dentistry.

## 5. Bioactive and Biologically Instructive Interfaces

One of the most important directions is changing the surface of an implant or other dental material from a passive boundary into an active interface capable of transmitting molecular signals locally. It is important to separate, on the one hand, the demonstrated functionality of a particular platform, and on the other, broader possibilities for “biological control” of tissue responses.

Valentino et al. [6] developed a titanium implant coating that enables local delivery of miR-21. Cationic lecithin- and polyethyleneimine-based nanoparticles were used as microRNA carriers, while a chitosan coating helped anchor the system to the implant surface and supported prolonged release. The study showed that miR-21 can be delivered into periodontal ligament cells while preserving biological activity. These findings should be viewed mainly as proof of concept: implant surfaces may be able to function as local molecular interfaces. Further evaluation of anti-inflammatory, osteogenic, and clinically relevant effects will require more complex models and in vivo validation.

A higher level of complexity is seen in nucleic-acid delivery systems designed for tissue specificity. Xu et al. [7] created lipid nanoparticles using microfluidic mixing for mRNA delivery and functionalized them with alendronate to enhance affinity for bone tissue. Optimized nanoparticles bound hydroxyapatite and bone, enabled cellular uptake, and supported effective mRNA translation; in vivo studies confirmed preferential accumulation in bone. While this work is not directly tied to dental technologies, it provides important supporting proof of principle that is relevant to future regeneration of alveolar and maxillofacial bone.

More broadly, approaches like these reinforce the concept of a biologically instructive interface. In this view, material performance depends not only on chemical composition, surface structure, and passive biocompatibility, but also on the ability to guide cellular, immune, inflammatory, or regenerative responses within appropriate spatial and temporal patterns.

## 6. Towards an Integrated Local Therapeutic System

Modern reviews on smart and stimulus-responsive materials show active development of platforms that respond to pH, enzymatic activity, reactive oxygen species, temperature, light, ultrasound, and also electrical and mechanical stimuli [8,9]. However, stimulus sensitivity alone does not create a new scientific paradigm. What matters more is the shift toward systems that connect pathological context, local delivery, quantitatively defined exposure, and measurable biological response.

For that reason, we suggest viewing the most promising design unit not simply as a “smart material,” but as a connected system: “local microenvironment—therapeutic platform—local exposure—biological response.” This chain can be expanded further with state measurement and feedback mechanisms, forming an adaptive therapeutic loop (Figure 1). This method moves attention away from maximizing a single material property and toward coordinating multiple features tied directly to the clinical goal.

From a pharmaceutical perspective, this means the therapeutic effect of a local system is determined not only by the administered or loaded dose, but—primarily—by the dynamic local exposure that forms. For hydrogels, coatings, nanoparticles, and other dental carriers, exposure depends on release, retention, transport, local clearance, penetration into tissue or biofilm, affinity to the target, and the current microenvironmental state. Therefore, comparing systems only by loading amount and release curves may be inadequate for predicting therapeutic effectiveness.

## 7. Key Translational Barriers

Despite major progress, these technologies share several challenges. First, there is a gap between laboratory models and the real clinical microenvironment. Standard cell culture systems and microbiological models only partially reflect the dynamics of saliva, gingival fluid, mechanical loading, multispecies microbiota, individual tissue characteristics, and repeated exposure cycles. A more rigorous model sequence is needed: moving from in vitro to ex vivo, then in vivo, and finally to clinically oriented validation.

Second, there is insufficient standardization. Differences in biofilm models, photodynamic therapy protocols, release-testing methods, and biological endpoints make it difficult to compare results across laboratories. For new technologies, standardization should cover not only material properties but also microenvironment parameters, exposure conditions, and the characteristics of the biological response.

Third, efficacy and safety must be assessed comprehensively. High antimicrobial activity should not be achieved at the expense of tissue damage or persistent disruption of normal microbiota. At the same time, high biological activity alone does not guarantee resistance to bacterial recolonization. For nanomaterials and nucleic acid delivery systems, studies of biodistribution, degradation behavior, immune response, and long-term safety are especially important.

Fourth, links between platform properties and real therapeutic exposure are still not strong enough. For most local systems, it is much easier to measure loading and drug release than to determine the actual drug concentration directly within target tissue, biofilm, or the periodontal pocket. Without this connection, it is difficult to translate findings rationally between models and to predict clinical outcomes.

Finally, the more complex the system becomes, the greater the translational burden. As materials or devices combine more functions, reproducibility of manufacturing, sterilization procedures, long-term stability, scalability, quality control, regulatory classification, and clinically meaningful efficacy criteria become increasingly critical.

## 8. Prospects: Five Stages of Transition to Adaptive Local Therapy

We believe future progress will be driven less by the emergence of a new class of “smart” dental materials and more by changes in the principles used to design local therapeutic systems.

1. **From static release to controlling local therapeutic exposure.** The goal should not be maximum drug loading, or even the longest release, but maintaining an effective and safe concentration in the target area for the required duration. This requires accounting for release, retention, transport, clearance, and tissue distribution processes.
2. **From simply antimicrobial activity to ecological control of dysbiotic biofilms.** The focus shifts from measuring overall reductions in microbial load to suppressing pathological biofilm organization, limiting reformation, and supporting restoration of stable “host–microbiome” homeostasis.
3. **From bioactive surfaces to biologically instructive interfaces.** The surface of an implant, a restorative material, or a regenerative matrix can become a local platform for delivering controlled anti-inflammatory, immunomodulatory, osteogenic, and other regulatory signals. In this kind of system, functionality depends not only on chemical composition and surface structure, but also on the ability to direct local biological responses.
4. **From empirical optimization to model-informed design.** Mechanistic modeling of local pharmacokinetics and pharmacodynamics makes it possible to connect carrier properties with exposure level and final therapeutic effect. Composition, geometry, degradation rate, and release profile can be optimized based on a predetermined therapeutic window rather than relying only on repeated iterative experiments.
5. **From open-loop therapy to context-dependent, adaptive systems.** Differences between individuals in salivary flow, gingival fluid composition, biofilm structure, inflammatory state, and local anatomy can strongly influence how well the same system performs. Incorporating these factors into mechanistic models creates a foundation for model-informed personalization. The next step may be feedback-based systems: sensors or multimodal diagnostics detect microenvironment status, quantitative models interpret the data, and local intervention parameters are adjusted according to the organism’s response [11].

The idea of closed intraoral therapeutic loops is starting to take shape technically. Modern integrated oral wearable devices combine continuous biosensing, wireless communication, and controlled drug delivery (or other therapeutic interventions), creating the prerequisites for closed-loop theranostic systems [11]. However, substantial barriers still need to be overcome before clinically mature solutions are possible, including long-term stability, power supply constraints, biocompatibility, data standardization, and clinical validation.

Within this framework, artificial intelligence and digital twins should mainly be treated as tools for integrating data and supporting modeling, rather than as end goals on their own. Digital twin technologies in dentistry are still in early development: most published work covers conceptual or prototype studies with limited clinical validation [12]. Their potential value is in combining patient-specific observations with mechanistic models of intervention and response, which could eventually help select and adapt local therapies.

Ultimately, the most promising design unit is not a single material or drug, but an integrated system that includes “microenvironment—platform—exposure—response—feedback.” This strategy connects materials science and pharmaceutics with microbiology, regenerative medicine, sensor technologies, and computational modeling, while also establishing measurable criteria for preclinical and clinical translation.

## 9. Conclusions

Two consecutive issues of this special edition reflect how approaches to developing dental biomaterials and therapeutic agents are evolving. The first issue highlighted a wide range of functional solutions, including nanomaterials and active substances, mineralization, regenerative strategies, anti-biofilm approaches, as well as prevention and treatment of oral diseases. The second issue demonstrates that further progress depends increasingly on integrating these functions and on being able to control where, when, and at what concentration therapeutic effects occur.

Therefore, the main challenge for the next stage is to move away from optimizing individual material features toward managing the entire chain: “microenvironment—therapeutic platform—local exposure—biological response.” Microsensitive materials, local delivery of regulatory molecules, biologically instructive interfaces, and mechanistic modeling are already forming key parts of such a system. Their integration with patient-specific data—and, in the future, with feedback mechanisms—could shift dental biomaterials from predetermined platforms toward adaptive local therapeutic systems.

We believe one of the most important criteria for next-generation dental biomaterials and delivery systems will be the ability to quantitatively connect material properties not only with release kinetics, but also with clinically meaningful spatiotemporal local exposure and the biological response it produces. This shift—from a material-centered approach to one guided by exposure and response data—is what will make the development of new dental materials more rational, more comparable, and more aligned with practical translation.

## Figures and Tables

**Figure 1:**
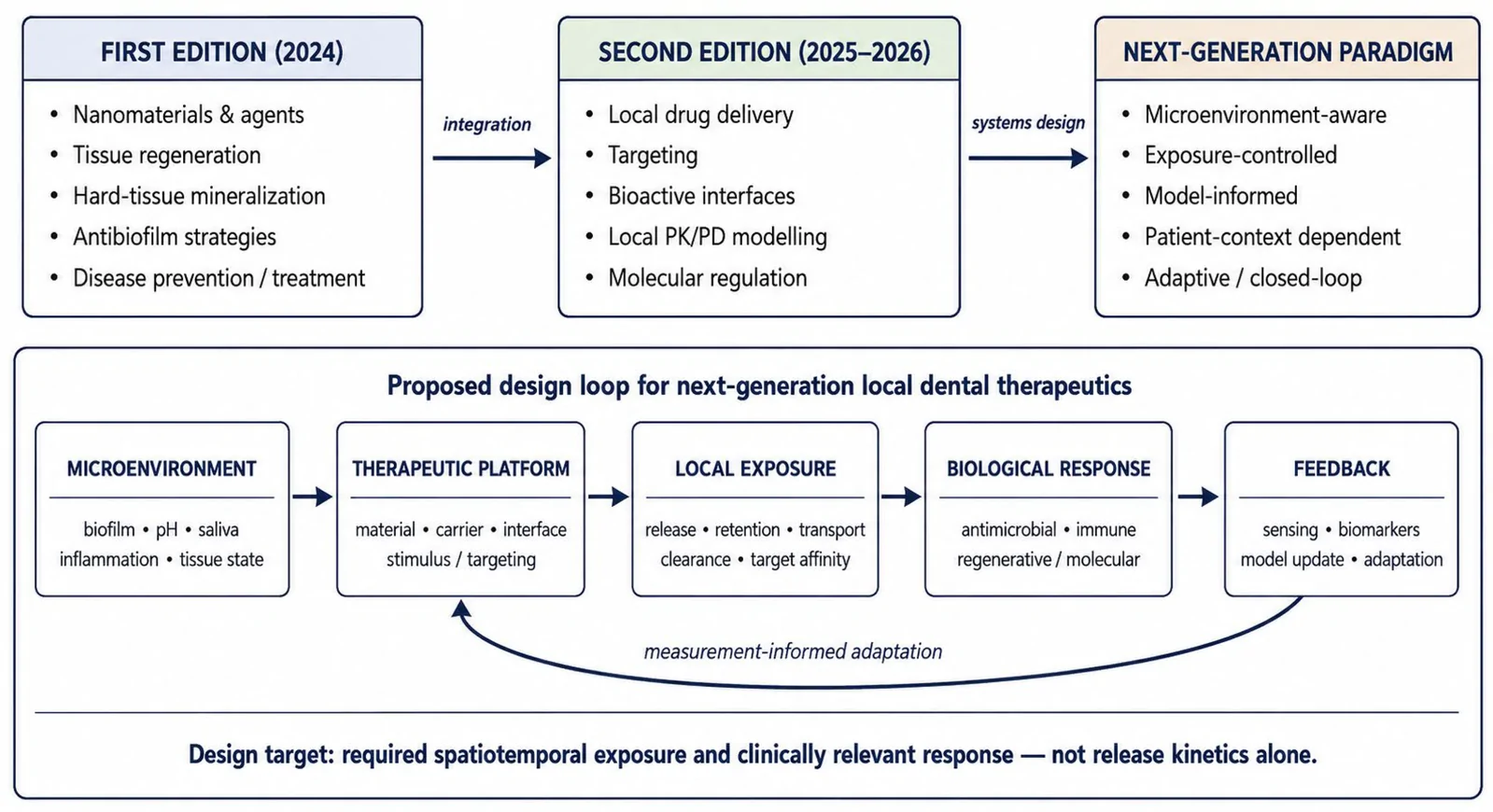
Conceptual evolution from functional dental biomaterials to adaptive local therapy. The first Special Issue covered the major functional directions; the second demonstrates how they integrate through local delivery, targeting, bioactive interfaces, and quantitative modeling. The proposed next paradigm treats therapy as a connected loop: “microenvironment—therapeutic platform—local exposure—biological response—feedback”.
